# A longitudinal investigation of the relationship between dimensional psychopathology, gray matter structure, and dementia status in older adulthood

**DOI:** 10.1017/S0033291724003490

**Published:** 2025-02-04

**Authors:** Nicholas Hoy, Monika Waszczuk, Matthew Sunderland, Samantha J. Lynch, Perminder S. Sachdev, Henry Brodaty, Simone Reppermund, Louise Mewton

**Affiliations:** 1 The Matilda Centre for Research in Mental Health and Substance Use, University of Sydney, Sydney, Australia; 2 Department of Psychology, Rosalind Franklin University of Medicine and Science, North Chicago, IL, USA; 3 Department of Psychiatry, Université de Montréal, Montreal, Canada; 4 Centre de recherche Azrieli du CHU Sainte-Justine, Montreal, Canada; 5 Centre for Healthy Brain Ageing, Discipline of Psychiatry and Mental Health, School of Clinical Medicine, University of New South Wales, Sydney, Australia

**Keywords:** psychopathology, transdiagnostic dimensions, gray matter structure, dementia status, older adulthood, longitudinal analysis

## Abstract

**Background:**

The structure of psychopathology can be organized hierarchically into a set of transdiagnostic dimensional phenotypes. No studies have examined whether these phenotypes are associated with brain structure or dementia in older adults.

**Methods:**

Data were drawn from a longitudinal study of older adults aged 70–90 years at baseline (*N* = 1072; 44.8% male). Confirmatory factor models were fit to baseline psychiatric symptoms, with model fit assessed via traditional fit indices, model-based reliability estimates, and evaluation of model parameters. Bayesian plausible values were generated from the best-fitting model for use in subsequent analyses. Linear mixed models examined intraindividual change in global and regional gray matter volume (GMV) and cortical thickness over 6 years. Logistic regression examined whether symptom dimensions predicted incident dementia over 12 years.

**Results:**

A higher-order model showed a good fit to the data (BIC = 28,691.85; ssaBIC = 28,396.47; CFI = 0.926; TLI = 0.92; RMSEA = 0.047), including a general factor and lower-order dimensions of internalizing, disinhibited externalizing, and substance use. Baseline symptom dimensions did not predict change over time in total cortical and subcortical GMV or average cortical thickness; regional GMV or cortical thickness in the frontal, parietal, temporal, or occipital lobes; or regional GMV in the hippocampus and cerebellum (all *p*-values >0.5). Finally, baseline symptom dimensions did not predict incident dementia across follow-ups (all *p*-values >0.5).

**Conclusions:**

We found no evidence that transdiagnostic dimensions are associated with gray matter structure or dementia in older adults. Future research should examine these relationships using psychiatric indicators capturing past history of chronic mental illness rather than current symptoms.

## Introduction

As the number and proportion of older adults continue to expand globally (World Health Organization, 2022), it is increasingly important to understand the mechanisms and processes that impact healthy aging in this population. In particular, novel approaches are needed to identify potential targets for the prevention of neurodegeneration and dementia in later life. An extensive body of research indicates that psychiatric disorders are associated with alterations in brain structure and function across the lifespan, including accelerated brain aging (Cole et al., 2019; Wrigglesworth et al., 2021). Several psychiatric disorders are also associated with a greater likelihood of dementia diagnoses in later life (Richmond-Rakerd et al., 2022) and genomic research indicates shared biological mechanisms between psychiatric and neurodegenerative diseases (including dementia; Wingo et al., 2022). These associations appear to cut across traditional diagnostic categories, with a range of putatively distinct psychiatric disorders being nonspecifically associated with both neurodegeneration and dementia risk. This raises the possibility that transdiagnostic models may hold more utility than traditional diagnostic categories in research aiming to disentangle the relationships between psychopathology, neurodegeneration, and dementia in later life.

### Transdiagnostic dimensional models of psychopathology

Transdiagnostic dimensional models of psychopathology have recently gained popularity as an alternative approach to the classification of mental illness (Kotov et al., 2017, 2020; Kotov et al., 2021; Krueger et al., 2021; Watson et al., 2022). In these models, psychiatric symptoms and traits are placed at the lowest level of a structural hierarchy and grouped together into higher-order dimensions (e.g., internalizing, externalizing) based on their patterns of covariance (Kotov et al., 2017, 2021). For example, the internalizing dimension captures covariation among emotional indicators of psychopathology (e.g., anxiety, depression), while externalizing captures more behaviorally focused indicators (e.g., disinhibition, aggression, substance use; Krueger et al., 2021; Watson et al., 2022). These phenotypes also tend to exhibit positive correlations with one another, suggesting the presence of a single superordinate dimension of psychopathology (i.e., general psychopathology; Kotov et al., 2021) This general dimension is argued to reflect a general underlying liability toward the full spectrum of mental illness (Caspi et al., 2014; Caspi & Moffitt, 2018a).

### Transdiagnostic dimensional models of psychopathology in neuroscientific research

The underlying neurobiology of mental illness is closely aligned with the structure of psychopathology identified through phenotypic research. For example, the neural correlates of specific psychiatric disorders are associated with subclinical symptom expression in general population samples, supporting the dimensionality of mental illness (Besteher et al., 2020). Meta-analytic evidence further indicates that abnormalities in both brain structure and function are largely shared across putatively distinct diagnostic categories (Goodkind et al., 2015; McTeague et al., 2017; Sha et al., 2019), consistent with the correlational structure of psychopathology identified through latent variable modeling. These findings indicate that the neural architecture underlying mental illness is poorly aligned with the discrete categorical boundaries of traditional classification systems. In contrast, transdiagnostic models directly estimate the observed dimensionality and correlational structure of psychopathology (e.g., comorbidity). The phenotypes derived from these models show greater validity and reliability than discrete (e.g., categorical) phenotypes, with the resulting increase in power substantially decreasing the need for larger sample sizes (Markon et al., 2011). The hierarchical structure of these models also allows researchers to investigate the neural correlates of psychopathology at different levels of specificity (i.e., the correlates of general and specific/lower-order symptom dimensions; Latzman & DeYoung, 2020; Zald & Lahey, 2017). An important advantage of this approach is that it allows for disentangling shared from unique associations, which would be otherwise obscured in case–control studies of individual psychiatric disorders. Given these advantages, the use of transdiagnostic dimensional models may facilitate discoveries with respect to the relationship between psychopathology and brain health in older adulthood.

However, a recent systematic review found that not a single study has investigated associations between brain structure and transdiagnostic symptom dimensions specifically in older adults (i.e., 60 years or older; Hoy et al., 2023). In younger samples, transdiagnostic symptom dimensions were consistently associated with pervasive alterations in gray matter structure across several studies (Hoy et al., 2023). For example, general and specific/lower-order dimensions (e.g., internalizing, externalizing) were associated with lower global measures of gray matter volume (GMV) and surface area in multiple studies spanning childhood to young adulthood (Kaczkurkin et al., 2018; Mewton et al., 2022; Parkes et al., 2021; Romer et al., 2023). These findings highlight the utility of dimensional models in psychiatric neuroscience, which has historically aimed to identify disorder-specific correlates within relatively discrete brain regions. Further research is needed to examine whether these phenotypes are also associated with reduced gray matter structure in older adulthood and to determine whether there is evidence of age-specific differences in the nature of these associations. In particular, establishing that these phenotypes can be used to predict change in gray matter structure over time in older adults would provide novel targets for the promotion of brain health in this population.

### Dimensional models of psychopathology as a novel framework for investigating the relationship between mental illness and dementia

An extensive body of evidence indicates that psychiatric illness is associated with cognitive decline and dementia risk in older adulthood. Several systematic reviews and meta-analyses have demonstrated a link between individual psychiatric disorders and dementia risk (Becker et al., 2018; Cai & Huang, 2018; Velosa et al., 2020). A recent population-based study of 1.7 million people also found that those with *any mental disorder* were significantly more likely to develop a dementia diagnosis in older adulthood (Richmond-Rakerd et al., 2022). This research suggests that psychopathology is nonspecifically associated with dementia risk; however, no studies have directly examined whether transdiagnostic dimensional phenotypes can be used to predict diagnoses of dementia in older adults. Determining whether these phenotypes can be used to predict diagnoses of dementia will provide important insights into the relationship between mental illness and one of the leading causes of burden of disease in older adulthood. Moreover, establishing the predictive utility of these phenotypes would facilitate the development of novel preventative strategies that target dimensional psychopathology while simultaneously reducing the risk of dementia in older adulthood.

### The current study

The current study aimed to determine whether transdiagnostic symptom dimensions can be used to predict intraindividual change in gray matter structure over 6 years of follow-up and incident dementia over 12 years of follow-up in older adults. The aims, research questions, and analytic plan were preregistered on Open Science Framework (OSF; https://rb.gy/1nz92g). For the primary analyses, it was hypothesized that higher severity of general and/or specific symptom dimensions at baseline would predict a greater decline in global cortical GMV, subcortical GMV, and cortical thickness across time. For secondary analyses, it was hypothesized that higher severity of general and/or specific symptom dimensions at baseline would predict a greater decline in regional GMV and cortical thickness across time. Finally, it was hypothesized that greater general and/or specific symptom dimensions would predict a greater likelihood of a dementia diagnosis at any wave.

## Methods

### Sample and study design

Data were drawn from the Sydney Memory and Ageing Study (MAS; Sachdev et al., 2010), a longitudinal study of community-dwelling older adults in Sydney, Australia. Participants were 1037 older adults aged between 70–90 years old (*M* = 78.84; SD = 4.82; 44.8% male) at baseline (Table 1). Participants were followed across seven waves of data collection, with assessments occurring every 2 years (alongside brief phone interviews in intervening years). Informants were recruited for the majority of participants (93.9%), provided that they had contact with the participant for at least 1 hour per week and could answer questions regarding their cognitive ability and daily functioning. Recruitment and study enrollment took place between September 2005 and November 2007. Inclusion criteria included the following: (1) aged between 70–90 years old; (2) living in the community; (3) able to speak/write in English; and (4) ability to consent. Exclusion criteria included the following: (1) previous dementia diagnosis or diagnosis of dementia after comprehensive in-study assessment at baseline; (2) psychotic symptoms, schizophrenia diagnoses, or bipolar diagnoses; (3) diagnosis of multiple sclerosis, motor neuron disease, developmental disability, or progressive malignancy; (4) medical or psychological conditions that prevent participation; or (5) a Mini-Mental State Examination (Folstein et al., 1975) score of <24 (adjusted for age, education, and non-English speaking background). The MAS sample and study design are described in detail elsewhere (Sachdev et al., 2010) and outlined in the Supplementary Material (Appendix A).

**Table 1.** Baseline sample characteristics for the full sample and the MRI subsample

	Full sample (*N* = 1037)		MRI subsample (*n* = 532)^a^	
Categorical covariates	*N*	%	*N*	%
Sex (male)	465	44.8	242	45.5%

Continuous covariates	Mean	SD	Mean	SD
Age (years/continuous)	78.84	4.82	78.41	4.68
Education (years/continuous)	11.60	3.47	11.80	3.60
Total GMV (mm^3^)	-	-	552,914.1	52,540.02
Average cortical thickness (mm)	-	-	2.43	0.11

Other characteristics	*N*	%	*N*	%
Race/ethnicity				
Caucasian	1016	98.4	517	97.2
Asian	10	1.0	9	1.7
Mixed	3	0.3	2	0.4
Other	4	0.4	2	0.4

Dementia Status	*N*	%	*N*	%
Dementia diagnosis^b^	269	25.9	-	-

*Note.* This table outlines the baseline characteristics for the full sample of participants from the Sydney Memory and Ageing Study (MAS) and the subsample of participants who completed MRI scanning at baseline.

a Follow-up MRI data were collected at Wave 2 (*n* = 417) and Wave 4 (*n* = 262).

b Dementia diagnosis data indicate the number of participants who received a diagnosis of dementia at *any follow-up wave.* Participants who received a diagnosis at one wave but not at subsequent waves were removed from the analysis (*n* = 7).

### Indicators of psychopathology

Indicators of psychopathology were derived from multiple self- and informant-report measures administered at baseline. The 15-item Geriatric Depression Scale (GDS) was designed to measure depressive symptoms over the past week in older adults (Yesavage et al., 1982). The Goldberg Anxiety Scale (GAS) is a 9-item measure of anxiety symptoms over the past month (Goldberg et al., 1988). The Kessler 10 (K10) is a 10-item measure of psychological distress over the past 30 days (Kessler, 1994). The Neuropsychiatric Inventory (NPI) assesses a range of psychiatric symptoms in people with dementia (Cummings et al., 1994), administered to informants of nondemented participants at baseline. The current study only included NPI items relating to agitation/aggression, irritability/lability, and disinhibition. Finally, substance use was measured via a combination of self-report items relating to alcohol and nicotine use. Items from these measures were included in subsequent latent variable models as indicators of latent internalizing (i.e., GDS, GAS, and K10 items), disinhibited externalizing (i.e., NPI screening items for agitation/aggression, disinhibition, and irritability/lability), and substance use (i.e., alcohol and nicotine use items). Further details of symptom-level indicators are included in all latent variable models and are provided in Appendix B and Supplementary Table S1. Tetrachoric correlations among those indicators are provided in Supplementary Table S2.

### Brain structural outcome measures

Details of the neuroimaging protocol are described in detail elsewhere (Sachdev et al., 2010) and outlined in the Supplementary Material (Appendix C). Briefly, all participants were invited to complete brain magnetic resonance imaging (MRI), and consenting participants were further screened for contraindications (i.e., pacemaker, metallic implant or foreign bodies, cochlear implants, ferromagnetic homeostatic clips, claustrophobia). Approximately half of the sample (50.75%) agreed to complete MRI scanning at baseline (*n* = 544). Following quality control procedures (Jiang et al., 2014) and exclusions due to medical issues that emerged after consenting to MRI scans (e.g., back problems), the final analytic sample size at baseline was *n* = 532. Follow-up MRI scans were also completed at Wave 2 (*n* = 417) and Wave 4 (*n* = 262). We conducted paired samples *t*-tests and chi-square tests to examine differences in covariates (i.e., age, sex, education, total GMV, average cortical thickness) between those with complete and incomplete MRI follow-up data (Table S3). Those with complete MRI data were significantly younger at baseline and had larger total GMV at baseline, compared to those with incomplete MRI data. The present study used preprocessed structural neuroimaging data (i.e., cortical and subcortical volume, cortical thickness). GMV and cortical thickness within 68 cortical regions and GMV within 19 subcortical regions (including the brain stem) were used to construct brain structural variables for primary and secondary outcomes. Primary outcomes included global measures of brain structure, that is, total cortical GMV, total subcortical GMV, and average cortical thickness. Secondary outcomes included 10 region-of-interest (ROI) measures, that is, total GMV and average cortical thickness in the frontal, parietal, temporal, and occipital lobes, as well as total GMV in the bilateral hippocampus and cerebellum. All brain structural variables were winsorized to be within ±3 standard deviations (SD) of the mean (*M*).

### Dementia status outcome

All participants were free of dementia at baseline. Dementia status was determined via consensus diagnosis from a multidisciplinary panel of experts at each wave of data collection, on the basis of available clinical, neuropsychological, laboratory, and neuroimaging data. Further details of the diagnostic procedures are described in detail elsewhere (Sachdev et al., 2010) and outlined in the Supplementary Material (Appendix D). For the current study, a single binary variable was used to indicate whether participants were diagnosed with dementia at *any follow-up wave* (across 12 years of follow-up). Participants coded as having dementia at one wave and no dementia at subsequent waves (*n* = 7) were removed from the analysis.

### Model estimation and assessment of model fit

The latent structure of psychopathology was examined using confirmatory factor analysis (CFA) of symptom-level categorical indicators of mental illness in the full sample at baseline. Four CFA models that are most commonly used to measure the latent structure of psychopathology were fit to the data (i.e., a one-factor model, a correlated-factors model, a bi-factor model, and a higher-order factor model). The use of confirmatory factor analytic models and allocation of indicators to specific/lower-order factors was based on extensive research detailing the latent structure of psychopathology (Caspi et al., 2014; Caspi & Moffitt, 2018b; Kotov et al., 2017, 2021; Krueger et al., 2021; Watson et al., 2022). The best-fitting factor model was selected for inclusion in subsequent analyses based on traditional fit indices, model-based estimates of reliability, and evaluation of model parameters (e.g., the significance, direction, and standard errors of the factor loadings). Details of model estimation and assessment of model fit are presented in the Supplementary Material (Appendices E–G) and examples of the Mplus code for each latent variable model are provided on OSF (https://osf.io/uhds9/).

### Bayesian plausible values

Bayesian plausible values (BPVs) were generated for each participant and each latent symptom dimension. BPVs are a *set* of factor scores derived from multiple imputations that provide more reliable estimates and address biases in measurement (Muthen & Asparouhov & Muthen, 2010). Calculating BPVs involves taking multiple random draws (i.e., imputations) from the posterior distribution of factor score estimates for each participant, providing a range of plausible values for a given factor score. For each participant, 100 plausible values were estimated for each latent factor (i.e., 100 imputed factor scores from the posterior distribution were estimated for general and specific/lower-order factors). BPV estimation was conducted in Mplus Version 8.10 (Muthén & Muthén, 2017). The 100 data sets were then analyzed simultaneously in R version 4.3.2 using (generalized) linear regression and (generalized) linear mixed models within a multiple imputation framework (mitml R package; Bates et al., 2015). Factor scores derived from CFA models provide a single-point estimate of psychopathology for a given symptom dimension. The distributions of these scores are highly skewed when relying on categorical indicators, as in the current study. These scores are also likely to contain substantial random error (i.e., factor indeterminacy; Wu, 2005); however, we were unable to directly calculate factor determinacy in the current study due to the inclusion of multiple dichotomous indicators (Beauducel & Hilger, 2017; Ferrando & Lorenzo-Seva, 2018; Forbes et al., 2021). In contrast, BPVs offer a less biased estimation of the population mean and variance of psychopathology by accounting for the uncertainty around factor scores through multiple imputations. An alternative approach would be to estimate associations simultaneously within a structural equation modeling framework; however, this was unable to be done in the current study due to model complexity.

### Analysis plan

Our primary analyses examined whether baseline general and specific/lower-order symptom dimensions predict intraindividual change in total cortical GMV, total subcortical GMV, and average cortical thickness across follow-up waves. Baseline BPVs for general and specific/lower-order symptom dimensions were entered as predictors in a series of linear mixed models with brain structural measures included as the outcome variable. All linear mixed models examined associations between one set of BPVs (e.g., for general psychopathology) and one brain structural variable (e.g., total GMV). Nesting of longitudinal measurements in participants was handled via the use of random intercepts and wave was represented as a categorical variable. All linear mixed models included sex, age, education, and MRI scanner as covariates. The primary estimate of interest was the wave by dimension interaction (e.g., wave by general psychopathology), which indicates whether there was an association between baseline symptom dimensions and change in outcomes over time. The following equation provides an example of the linear mixed models used to estimate wave x dimension interactions:


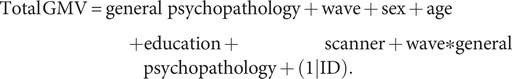


Secondary analyses examined whether baseline general and specific/lower-order symptom dimensions predict intraindividual change in regional measures of GMV and cortical thickness across follow-up waves. Specific outcome measures included: total GMV and average cortical thickness in the frontal, parietal, temporal, and occipital lobes, as well as total GMV in the bilateral hippocampus and cerebellum. These analyses followed the same methodology as for primary outcomes. All linear mixed models included sex, age, education, MRI scanner, and either total GMV or average cortical thickness as covariates. Additional secondary analyses examined whether baseline general and specific/lower-order symptom dimensions predict dementia status across 12 years of follow-up. Baseline BPVs were entered separately as predictors in a series of logistic regression models, with dementia status at any wave included as a binary outcome variable. All analyses were run over 100 imputations and the results were pooled into a final set of estimates within a multiple imputation framework. Missing data were handled via Full Information Maximum Likelihood (FIML) in Mplus. Benjamini–Hochberg false discovery rate (FDR) correction was used to correct for multiple testing, with an FDR threshold of 5% (*α* = 0.05; Appendix H). Examples of the R code used to conduct these analyses are provided on OSF (https://osf.io/uhds9/). There were two minor deviations from the preregistered analysis, which are outlined in Appendix I.

### Post-hoc analyses

Most research investigating the neural correlates of transdiagnostic symptom dimensions has been conducted cross-sectionally in samples of youth. As such, post-hoc analyses examined whether general and specific/lower-order dimensions predict baseline measures of gray matter structure in older adulthood. Baseline BPVs for general and specific/lower-order symptom dimensions were entered separately as predictors in a series of linear regression models, with baseline measures of GMV and cortical thickness included as the outcome variables. These analyses examined associations with the same brain structural measures (i.e., global and regional) included in primary and secondary analyses. All analyses included sex, age, education, and MRI scanner as covariates. Analyses of regional brain structure included additional controls for either total GMV or average cortical thickness. All analyses (i.e., primary, secondary, and post-hoc) of regional gray matter structure were also re-run without controlling for total GMV or average cortical thickness in order to examine both absolute and relative effects. Finally, we ran a series of unconditional linear mixed models (i.e., without predictors included) to examine the trajectories of each brain structural outcome measure over time (Appendix J). For all post-hoc analyses, Benjamini–Hochberg FDR correction was used to correct for multiple comparisons, with an FDR threshold of 5% (*α* = 0.05).

## Results

### Structural validity of latent variable models

**Figure 1.**
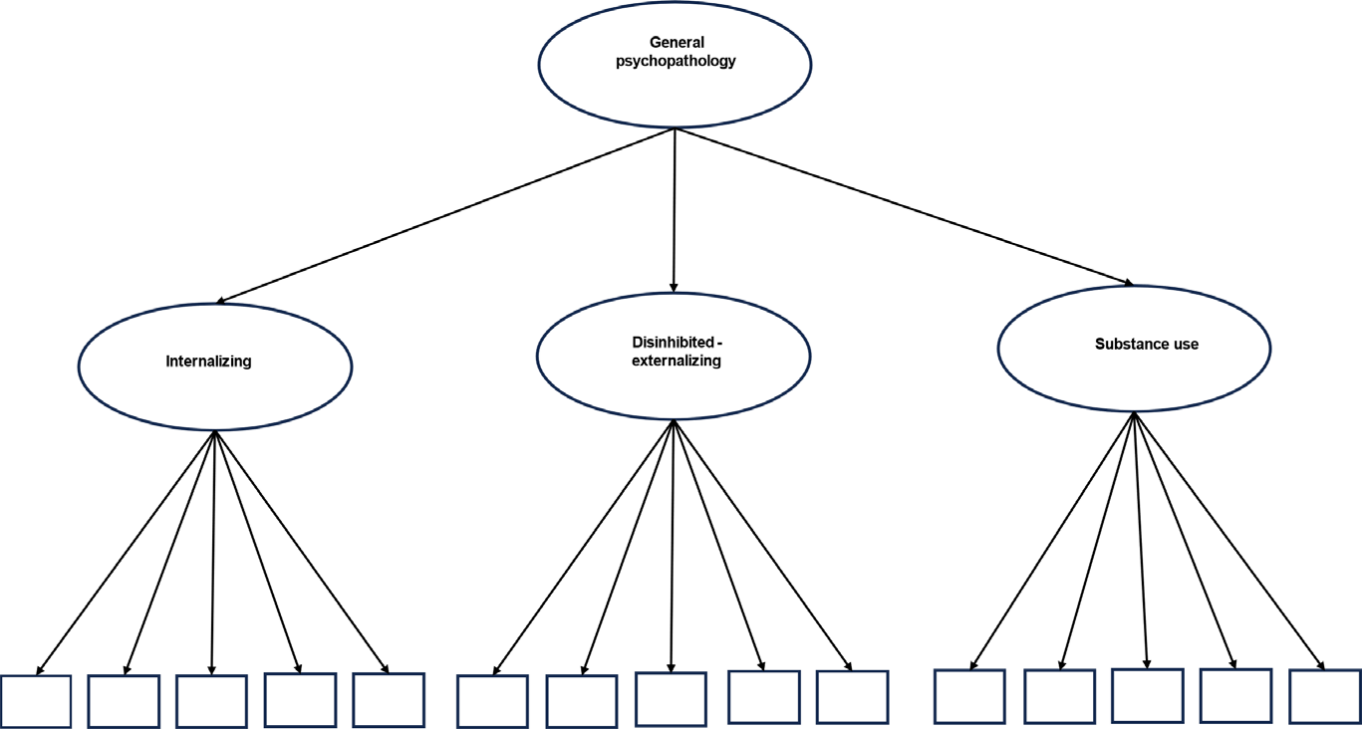
Figure representing the hierarchical structure of psychopathology in the Sydney MAS sample. *Note.* Sydney MAS, Memory and Ageing Study. This figure outlines the higher-order confirmatory factor model that was derived from symptom-level indicators of psychopathology at baseline and subsequently included in all primary, secondary, and post-hoc analyses. In this model, observable indicators are specified to load onto one of three specific factors (labeled internalizing, disinhibited externalizing, and substance use), and these factors are specified to load onto a single higher-order general dimension of psychopathology. Latent symptom dimensions are depicted using circles and observable indicators of psychopathology are depicted using squares.

Traditional model fit statistics for the four CFA models are provided in Table S4 and model-based estimates of reliability are provided in Table S5. The best-fitting model based on traditional fit statistics (i.e., BIC, ssaBIC, CFI, TLI, and RMSEA values) was the bi-factor model. However, bi-factor models have a tendency to provide a better fit than competing models when relying solely on traditional fit statistics and there is growing interest in the use of alternative approaches to model selection (Forbes et al., 2021; Watts et al., 2019). The higher-order model was superior in terms of model-based estimates of reliability (i.e., ECV, PUC, Omega H/HS values) and evaluation of model parameters. The higher-order model (Figure 1) was selected for inclusion in subsequent analyses, based on: (1) evaluation of standardized factor loadings (i.e., all positive in direction and significant for the higher-order model); (2) lower standard errors of the factor loadings (i.e., more precise estimates of these parameters); (3) evidence of multidimensionality yet poor reliability of general and specific factors of the bi-factor model based on model-based reliability coefficients (i.e., ECV, PUC, Omega H/HS values); and (4) evidence of greater construct reliability and replicability of specific factors (i.e., greater H values). For the higher-order model estimated using WLSMV, the disinhibited-externalizing factor loaded most strongly on the general factor (0.574), followed by internalizing (0.368), and substance use (0.322). These factor loadings are consistent with those of the higher-order model estimated using MLR (disinhibited externalizing = 0.55; internalizing = 0.375; substance use = 0.356). Model selection procedures are detailed extensively in the Supplementary Material (Appendices E–G). Standardized factor loadings and standard errors for all latent variable models (run using MLR and WLSMV estimation) are presented in Supplementary Tables S6–S13. Given inconsistent conclusions depending on approaches to model selection (e.g., model fit statistics v. model-based estimates of reliability), additional sensitivity analyses were conducted by re-running all models (from primary, secondary, and post-hoc analyses) using the bi-factor model to generate BPVs.

### Primary outcomes

Table 2 presents the results of analyses examining whether latent dimensions of baseline psychopathology derived from a higher-order factor model predict variations in global measures of brain structure across time. There was little evidence that general and lower-order dimensions of psychopathology at baseline were associated with a change in total cortical GMV, total subcortical GMV, or average cortical thickness across subsequent waves. Standardized results for analyses of global brain structure are presented in Supplementary Table S14.

**Table 2.** Results from analyses examining whether transdiagnostic symptom dimensions derived from a higher-order factor model predict global measures of gray matter structure

	Total cortical volume				Total subcortical volume				Average cortical thickness			
	*β*	SE	95% CI	*p*	*β*	SE	95% CI	*p*	*β*	SE	95% CI	*p*
GP												
BL model	−1578	1823.333	−5157.59, 2002.51	0.387	−2196	787.472	−1767.02, 1327.79	0.780	−0.002	0.006	−0.01, 0.01	0.798
LMM												
GP*Wave2	9187	1265.720	−2393.52, 2577.27	0.942	1093	538.220	−948.01, 1166.54	0.839	−0.001	0.004	−9082.25, 0.007	0.850
GP*Wave4	3804	1646.124	−2855.91, 3616.66	0.817	1223	658.623	−1172.12, 1416.72	0.853	−0.003	0.005	−126.51, 0.007	0.606
INT												
BL model	−3319	1469.747	−6200.30, −438.25	0.024	2278	601.183	−1155.71, 1201.27	0.970	−0.005	0.005	−0.01, 0.004	0.318
LMM												
INT*Wave2	−1721	985.085	−2103.10, 1758.84	0.861	−1696	412.369	−978.03, 638.75	0.681	0.001	0.003	−5333.31, 0.007	0.784
INT*Wave4	1866	1245.753	−576.79, 4308.87	0.134	9701	498.514	−880.33, 1074.35	0.846	−0.002	0.004	−9746.73, 0.005	0.587
DEXT												
BL model	−5992	1378.737	−3305.43, 2107.04	0.664	−1569	604.027	−1343.60, 1029.88	0.795	−0.000	0.005	−0.009, 0.009	0.990
LMM												
DEXT*Wave2	5446	978.383	−1866.16, 1975.08	0.956	1988	415.050	−616.28, 1013.84	0.632	−0.000	0.003	−0.006, 0.006	0.980
DEXT*Wave4	1512	1240.205	−2285.78, 2588.09	0.903	1632	497.070	−813.25, 1139.57	0.743	−0.001	0.004	−0.008, 0.006	0.705
SUB												
BL model	−1974	1570.047	−5051.80, 1103.81	0.209	−4528	655.149	−1737.33, 831.79	0.490	−0.001	0.005	−0.01, 0.009	0.863
LMM												
SUB*Wave2	6314	1057.530	−1441.56, 2704.38	0.550	−9505	434.037	−945.84, 755.74	0.827	−0.004	0.003	−103.33, 0.003	0.287
SUB*Wave4	4447	1264.064	−2922.62, 2033.21	0.725	−5759	526.833	−1033.39, 1032.23	0.999	−0.005	0.004	−130.81, 0.003	0.223

*Note.* BL, baseline; LMM, linear mixed models; GP, general psychopathology; INT, internalizing; DEXT, disinhibited externalizing; SUB, substance use. BL Model refers to linear regression models predicting baseline GMV. LMM refers to linear mixed models predicting intraindividual change in GMV across waves. In all models, pooled estimates of multiply imputed general and specific factor scores were entered as predictors. All models controlled for age, sex, education, and MRI scanner. All p-values are prior to false discovery rate (FDR) correction, with bold text indicating significant associations. No results were significant after FDR correction.

### Secondary outcomes

The results for all secondary outcome measures are presented in Tables S15–S18. Pooled estimates of the BPVs for general and lower-order dimensions were not associated with a change in GMV across time in any cortical or subcortical ROI (Tables S15 and S17). Substance use was associated with increased cortical thickness over time within the parietal lobe at Wave 2 (*β* = 0.006; SE = 0.003; *p* = 0.049); however, this association did not survive FDR correction (Table S16). No other symptom dimensions were associated with regional cortical thickness over time. BPVs for general and lower-order factors were also not significantly associated with dementia status across waves (Table S18).

### Post-hoc analyses

Post-hoc analyses revealed no evidence of association between general psychopathology and total cortical GMV, total subcortical GMV, or average cortical thickness at baseline (Table 2). Internalizing was negatively associated with total cortical GMV (*β* = −3319; SE = 1469.747; *p* = 0.024) but not total subcortical GMV or average cortical thickness at baseline; however, this association did not survive FDR correction. Disinhibited externalizing and substance use factors were not associated with any global measure of gray matter structure at baseline. When controlling for global effects (i.e., total GMV, average cortical thickness), general and lower-order factors were not associated with baseline GMV or cortical thickness in any ROI (Tables S15–S17). Internalizing was significantly negatively associated with baseline GMV in the bilateral frontal lobe (*β* = −1332; SE = 585.206; *p* = 0.023) and bilateral temporal lobe (*β* = −8305; SE = 375.023; *p* = 0.027) when not controlling for total GMV; however, neither association survived FDR correction (Table S15). Disinhibited externalizing and substance use factors were not associated with baseline GMV or cortical thickness in any ROI when controlling or not controlling for global effects (Tables S15–S17). To examine the extent to which education might be driving our results, all analyses for the higher-order model were re-run without including education as a covariate. Before FDR correction, we found a significant negative effect of BPVs for the substance use factor on GMV within the occipital lobe (*β* = −408.499; SE = 206.464; *p* = 0.048) when not controlling for total GMV. All other outcomes were consistent with our initial analyses, suggesting that education did not confound the relationship between dimensions of psychopathology and gray matter structure in the current study. Results from post-hoc analyses using BPVs generated from a bi-factor model were consistent with those found for the higher-order model and are outlined in the Supplementary Material (Appendix K, Tables S19–S22). It should be noted that the results from analyses using BPVs generated for the bi-factor model are unlikely to be informative, given the problems evident in the factor loadings (e.g., the substantial number of factor loadings that were nonsignificant, negative in direction, and/or small in magnitude; Appendix G).

## Discussion

This study examined associations between latent transdiagnostic dimensions of psychopathology, gray matter structure, and dementia status in older adults. Consistent with previous research (Kotov et al., 2017, 2021), our confirmatory factor models demonstrated that psychopathology in older adulthood can be organized hierarchically into a set of general and specific/lower-order transdiagnostic symptom dimensions. However, no associations between these dimensions and changes in brain structure remained after FDR correction. Specifically, we found no evidence that baseline estimates of general and lower-order symptom dimensions predicted intraindividual change in global or regional gray matter structure across time. Our post-hoc analyses found no evidence of an association between transdiagnostic symptom dimensions and baseline measures of global and regional gray matter. There was also no evidence that general and lower-order dimensions predicted incident dementia, across 12 years of follow-up.

### Strengths and limitations

There are several strengths and limitations to the current study that are important to consider. Firstly, our study included a large sample size and repeated measurements of both brain structure (over 6 years of follow-up) and consensus diagnoses of dementia (across 12 years of follow-up). That said, future research would benefit from examining potential relationships with other neuroimaging measures (e.g., of white matter microstructure, functional connectivity) and more nuanced examination of dementia (e.g., specific subtypes rather than a general binary outcome measure). In addition, our study used a rigorous and theory-driven approach to modeling the latent structure of psychopathology. However, our measurement was somewhat limited by the lack of detailed psychiatric assessment in our data set. We were restricted to modeling internalizing and two subdimensions of externalizing because we did not have enough indicators to specify more commonly studied dimensions (e.g., broad externalizing, thought disorder). In addition, while there were a large number of indicators for internalizing there were substantially fewer indicators for the other lower-order factors. Our disinhibited-externalizing factor was defined by only three indicators (all informant-report items from the NPI) and our substance use factor was defined entirely by indicators of alcohol and nicotine use (as illicit substance use is uncommon in older adults). These limitations impact the extent to which we can compare our results to those found in younger samples. Future research would benefit from investigating these relationships using dimensional models derived from a more extensive set of psychiatric indicators.

It is also important to consider the selection criteria of the Sydney MAS when interpreting our results. While participants with mild cognitive impairment were eligible for inclusion and represented 36.7% of the sample at baseline (Tsang et al., 2013), those diagnosed with dementia or who scored below 24 on the Mini-Mental State Examination were excluded. This has the advantage of reducing potentially confounding effects of dementia and significant cognitive impairment, allowing for clearer examination of the extent to which psychopathology contributes to these outcomes in an otherwise healthy sample of older adults. However, these selection criteria also limit the representativeness of the Sydney MAS sample (Sachdev et al., 2010; Tsang et al., 2013). It is possible that these criteria selected for participants with a lower range of structural brain changes over time and a lower incidence of later onset dementia compared to the general population of those aged 70 years or older. The MAS sample is also relatively well educated (average education = 11.6 years) and not racially diverse (98.4% Caucasian), further limiting the generalizability of our results. Future research may therefore benefit from investigating these relationships in a more representative sample of older adults. However, few available large-scale longitudinal studies in community-dwelling older adults include detailed psychiatric assessment, as well as neuroimaging and dementia status data.

### The neural correlates of transdiagnostic symptom dimensions in older adulthood

The lack of significant associations between symptom dimensions and gray matter structure in the current study is inconsistent with findings in younger samples. Several cross-sectional studies have reported that general psychopathology, internalizing, and externalizing are associated with lower global and regional measures of gray matter structure from childhood to young adulthood (Kaczkurkin et al., 2019; Mewton et al., 2022; Parkes et al., 2021; Romer et al., 2023). These studies capture a critical period in which the brain undergoes substantial structural changes, with cortical thickness peaking in childhood and decreasing from childhood to adolescence and surface area peaking in preadolescence and decreasing slowly from adolescence to early adulthood (Tamnes et al., 2017; Wierenga et al., 2014). The majority of psychiatric disorders also tend to emerge between childhood and young adulthood (Solmi et al., 2022), perhaps driven by disruptions to normative maturational processes in the brain during this highly sensitive period of neurodevelopment. In contrast, the clinical picture of psychopathology in older adulthood may reflect: (1) symptoms that emerge early in development and persist or re-occur across the lifespan; (2) symptoms that first emerge in older adulthood; or (3) symptoms that specifically precede or follow from the onset of cognitive decline and dementia. Psychiatric symptoms that emerge in later life may be driven more strongly by environmental factors and physical comorbidities than genetic influences, which may exert less of an impact on brain structure. In the present study, our measurement models predominately included indicators of current symptom expression and may therefore be capturing late onset psychopathology. Future research should examine whether the relationship between transdiagnostic symptom dimensions and brain health in older adulthood differs as a function of age at symptom onset. Alternatively, potential associations between psychopathology and brain structure may be obscured by the impacts of age-related pathologies and neurodegeneration that emerge specifically in older adulthood. In either case, the inconsistency in results between our study and studies of younger samples underscores the importance of investigating these relationships across different age groups and highlights the complexities of doing so specifically in older populations. It is also important to consider sample size limitations when interpreting the lack of significant associations found for our longitudinal analyses of gray matter structure. MRI data were only available in a subsample of participants at baseline (*n* = 532), with substantial attrition across waves (*n* = 417 at Wave 2 and *n* = 262 at Wave 4). As such, it is possible that our analyses were not adequately powered to detect the effects of dimensional psychopathology on within-person changes in brain structure over time. This limitation was unavoidable given that our study relied upon secondary analysis of existing data and that there are few other large-scale studies of older adults that include the data necessary to address our research questions (i.e., broad measurement of psychopathology, repeated MRI measures). Furthermore, there were significant differences between those with complete versus incomplete follow-up MRI data. Specifically, those with complete MRI data were younger and had larger total GMV at baseline. As noted, age was included as a covariate in all analyses to control for age-related variation in GMV and missing data on the outcome was handled using maximum likelihood within a mixed model framework, which is more valid than complete case analysis (Dong & Peng, 2013). However, the overrepresentation of participants with greater baseline GMV in the follow-up sample may have reduced variability in GMV change, further limiting statistical power to detect associations with psychopathology dimensions. Additionally, since participants with higher baseline GMV may experience a different rate of decline than those with lower baseline GMV, our findings might not fully capture the broader relationship between psychopathology and intraindividual change in GMV over time in older adulthood.

### Transdiagnostic symptom dimensions as predictors of incident dementia in older adulthood

We found no evidence that general and specific/lower-order transdiagnostic symptom dimensions predict incident dementia. These findings are somewhat surprising given extensive evidence that dementia is associated with a range of psychiatric disorders (Becker et al., 2018; Cai & Huang, 2018; Mo et al., 2023; Richmond-Rakerd et al., 2022; Velosa et al., 2020). In the MAS sample specifically, previous studies have shown that baseline symptoms of depression, anxiety, apathy, and agitation are associated with mild cognitive impairment (Brodaty et al., 2012; Shahnawaz et al., 2013). However, the only indicators of psychopathology that have been found to predict incident dementia at follow-up in this sample are depressive symptoms (Brodaty et al., 2012). It may be that the relationship between current psychopathology and dementia risk is driven by specific symptoms (e.g., depressive symptoms) rather than transdiagnostic dimensions, perhaps indirectly through their association with certain physiological mechanisms and processes (e.g., increased cortisol levels, vascular risk factors, neuroinflammation) that are also implicated in dementia (Bennett & Thomas, 2014). Indeed, depressive symptoms are highly correlated with many other forms of psychopathology, which might account for the observed associations between dementia and a range of psychiatric disorders (Mo et al., 2023; Richmond-Rakerd et al., 2022). That said, further research is needed to thoroughly examine whether transdiagnostic symptom dimensions can be used to predict incident dementia in older adults. As noted, psychopathology in older adulthood may reflect symptoms that emerged earlier in development or had their onset in later life. These presentations likely follow distinct etiological pathways and may confer different risks with respect to the onset of dementia in older adulthood. For example, transdiagnostic dimensions derived from symptoms that were present earlier in development may be more likely to predict incident dementia due to their longer-term impacts on brain health and other related risk factors that unfold across the lifespan. Transdiagnostic symptom dimensions may also show greater predictive utility for specific subtypes of dementia (e.g., those characterized by psychiatric and behavioral disturbances, such as frontotemporal dementia) than for general measures of dementia status. There may also be a threshold effect in which dementia is transdiagnostically associated with clinically significant psychopathology but not with subthreshold symptom dimensions derived from general population samples, as in the current study. Finally, future research should also investigate the predictive utility of other symptom dimensions that are commonly investigated in younger samples (e.g., broad externalizing), which may show stronger associations with dementia.

## Conclusions

This is the first study to investigate the relationships between transdiagnostic symptom dimensions, brain structure, and dementia status in older adulthood. We found no evidence that transdiagnostic symptom dimensions are associated with gray matter structure or dementia status in this population. However, given that our current understanding of the neural correlates of transdiagnostic symptom dimensions comes almost exclusively from studies of youth, this study represents an important first step in determining the nature of these associations in an important and understudied age group. Future research would benefit from investigating these relationships in older adults using dimensional models derived from a more detailed set of psychiatric indicators. In addition, future studies should investigate whether age of symptom onset, normative brain aging, and age-related pathologies, impact the relationship between transdiagnostic symptom dimensions, brain structure, and dementia risk in later life.

## Supporting information

Hoy et al. supplementary material Hoy et al. supplementary material

## Supplementary material

The supplementary material for this article can be found at http://doi.org/10.1017/S0033291724003490.
