# Supplementary material for: A longitudinal investigation of the relationship between dimensional psychopathology, gray matter structure, and dementia status in older adulthood
Source: Psychol Med. 2025 Feb 4;55:e5. doi: 10.1017/S0033291724003490 (PMC11968117; doi:10.1017/S0033291724003490)
Supplement: Hoy et al. supplementary material [file S0033291724003490sup001.docx]

**A longitudinal investigation of the relationships between dimensional psychopathology, grey matter structure, and dementia status in older adulthood**

**Appendix A. Study design and sample**

Data were drawn from the Sydney Memory and Ageing Study (MAS; Sachdev et al., 2010), a longitudinal study of community-dwelling older adults. Participants were recruited from the electoral role of two federal government areas in Sydney, New South Wales, Australia (i.e., Kingsford-Smith and Wentworth). It is compulsory to register on the electoral role in Australia and registration data are publicly available. Prospective participants were first contacted via post and invited to participate if they were aged between 70-90 years old. Those who expressed an interest in the study were contacted via telephone and further assessed for eligibility. Informants were also recruited for the majority of participants (93.9%), provided that they had contact with the participant for at least one hour per week and were able to answer questions regarding their cognitive ability and daily functioning. Recruitment and study enrollment took place between September 2005 and November 2007. The final sample included 1037 older adults aged between 70-90 years old (M=78.84; SD=4.82; 44.8% male) at baseline. Study participants were followed across seven waves of data collection, with assessments taking place every two years (alongside brief phone interviews in intervening years). Inclusion criteria were: 1) aged between 70-90 years old; 2) living in the community; 3) able to speak and write in English; and 4) able to consent to participation. Exclusion criteria were: 1) previous diagnosis of dementia or received a diagnosis of dementia after comprehensive in-study assessment at baseline; 2) symptoms of psychosis, diagnosis of schizophrenia, or diagnosis of bipolar disorder; 3) a diagnosis of multiple sclerosis, motor neuron disease, developmental disability, or progressive malignancy; 4) medical or psychological conditions that prevent study participation; or 5) a Mini-Mental State Examination (MMSE; Folstein et al., 1975) score of < 24 (adjusted for age, education, and non-English speaking background).

**Appendix B. Indicators of psychopathology included in latent variable models.**

Indicators of psychopathology were derived from multiple self- and informant-report measures administered at baseline. The 15-item Geriatric Depression Scale (GDS) was designed to measure depressive symptoms over the past week in older adults (Yesavage et al., 1982). The Goldberg Anxiety Scale (GAS) is a 9-item measure of anxiety symptoms over the past month (Goldberg et al., 1988). Items 5-9 of the GAS were only asked of participants who endorsed at least two of the first four GAS items and were therefore not included in subsequent latent variable models. The Kessler 10 (K10) is a 10-item measure of psychological distress over the past 30 days (Kessler, 1994). The Neuropsychiatric Inventory (NPI) assesses a range of psychiatric symptoms in people with dementia (Cummings et al., 1994), administered to informants of non-demented participants at baseline. The current study only included NPI items relating to agitation/aggression, irritability/lability, and disinhibition. Finally, substance use was measured via a combination of self-report items relating to alcohol and nicotine use (see Table S1). Alcohol items included past-year measures of frequency (i.e., how often have you had a drink containing alcohol?), heavy consumption (i.e., how often do you have six or more standard drinks on one occasion?), memory problems (i.e., how often have you been unable to remember what happened to you the night before because of drinking?) and concerns from others (i.e., has a relative, friend, or a doctor or other health worker been concerned about your drinking or suggested you cut down?). Nicotine items included frequency of use per day (i.e., average number of cigarettes per day while smoking) and age of initiation (i.e., how old were you when you started smoking?). Items from each of these scales were included in subsequent latent variable models as indicators of latent internalizing (i.e., GDS, GAS, and K10 items), disinhibited externalizing (i.e., NPI screening items for agitation/aggression, disinhibition, and irritability/lability), and substance use (i.e., alcohol and nicotine use items). After running the initial latent variable models, certain items from the GDS and K10 were found to have zero cells in bivariate correlation tables with substance use and NPI items. As the items used to capture internalizing exceeded those of the other latent factors, items from the GDS and K10 were dropped in order to preserve the number of indicators for disinhibited-externalizing and substance use factors (see Table S1). Further details of symptom-level indicators included in all latent variable models and tetrachoric correlations among those indicators are provided in supplementary Tables S1-S2.

**Appendix C. Imaging procedure**

Imaging data were acquired using a Philips 3T Intera Quasar scanner and a Philips 3T Achieva Quasar Dual Scanner, with both scanners set to the same parameters (all analyses of brain structure included scanner type as a covariate). The Sydney Memory and Ageing Study (MAS) followed a standard imaging protocol, including: 1) a scout mid-sagittal cut for AC-PC plane alignment; and 2) 3D T1-weighted structural (T1w TFE – turbo field echo) MRI, acquired coronally with repetition time TR = 6.39 ms, echo time TE = 2.9 ms, flip angle = 8◦, matrix size = 256×256, field of view FOV = 256×256×190 mm3, and slice thickness = 1 mm with no gap between; yielding 1×1×1 mm3 isotropic voxels (Sachdev et al., 2010). The FreeSurfer (v7.1.0) processing pipeline was applied to T1-weighted scans for brain tissue segmentation (Fischl, 2012). The brain was segmented into 34 cortical regions per hemisphere and 9 subcortical regions per hemisphere (as well as the brain stem), using the Desikan–Killiany Atlas (Desikan et al., 2006).

**Appendix D. Dementia diagnoses**

Dementia status was determined via consensus diagnosis at each wave (Sachdev et al., 2010). Participants were assessed at fortnightly case conferences if they met the following criteria: 1) scored ≤ 1.5 standard deviations below normative data on a memory and non-memory measure; 2) scored ≤ 1.5 standard deviations below normative data on two non-memory measures; 3) showed reduced neuropsychological scores and a decline in informant-reported activities of daily living. Diagnoses were made by an expert panel of neuropsychiatrists, psychogeriatricians, and neuropsychologists based on thorough evaluation of relevant data (e.g., clinical, neuropsychological, laboratorial, and imaging data). Diagnoses were made according to DSM-4 criteria at each wave and by DSM-4 and DSM-5 criteria at waves six and seven; however, the present study only used diagnoses according to DSM-4 criteria in order to maintain consistency in measurement across follow-ups.

**Appendix E. Model Estimation**

The latent structure of psychopathology was examined using confirmatory factor analysis (CFA) of symptom-level categorical indicators of mental illness at baseline. Four CFA models that are most commonly used to measure the latent structure of psychopathology were fit to the data. This included a one-factor model, a correlated-factors model, a bi-factor model, and a higher-order factor model. For the one-factor model, all observed indicators of psychopathology were specified to load onto a single general factor of psychopathology. For the correlated factors model, observed indicators of psychopathology were specified to load onto three correlated factors (labelled internalizing, disinhibited-externalizing, and substance use). For the bi-factor model, observed indicators were specified to load onto a single general factor (labelled general psychopathology) and on one of three orthogonal (i.e., uncorrelated) specific factors (labelled internalizing, disinhibited-externalizing, and substance use). For the higher-order factor model, observed indicators were specified to load onto one of three specific factors (labelled internalizing, disinhibited-externalizing, and substance use) and these factors were specified to load onto a single higher-order general dimension of psychopathology. All models were estimated using the weighted least squares mean variance (WLSMV) and robust maximum likelihood (MLR) estimators in Mplus version 8.10 (Muthén & Muthén, 2018). For all models, the first factor loading was freely estimated and the means and variances of the latent factors were fixed to 0 and 1, respectively (i.e., standardized).

**Appendix F. Assessment of Model Fit**

Latent variable models were first estimated using the MLR estimator in Mplus (Muthén & Muthén, 2017). Models were directly compared using the Bayesian information criterion (BIC) and sample size adjusted BIC (ssaBIC). For both criteria, lower values indicate better model fit (Raftery, 1995). Models were also run using the WLSMV estimator to allow for assessment of absolute and incremental model fit, including the root mean square error of approximation (RMSEA; values < .05 indicating good model fit), the comparative fit index (CFI; values > 0.9 indicating good model fit), and the Tucker-Lewis index (TLI; values > 0.9 indicating good model fit). Additional model-based indices of reliability were calculated for the bi-factor model (using standardized factor loadings; Dueber, 2017), including the explained common variance (ECV), omega hierarchical (ωH) and omega hierarchical subscale (ωHS), and the percent uncontaminated correlations (PUC). ECV indicates the proportion of common variance in indicators that is explained by the general factor compared to the specific factors (Reise et al., 2010). Values range from 0-1, with values > 0.7 indicating an acceptable general factor and > 0.85 indicating that common variance is ‘essentially unidimensional’ (Rodriguez et al., 2016b). Omega hierarchical (ωH) and omega hierarchical subscale (ωHS) indicates the proportion of variance in total scores accounted for by general and specific factors after removing the effects of other factors, with values > 0.8 generally considered acceptable (Rodriguez et al., 2016a). Percent uncontaminated correlations (PUC) indicates the extent to which correlations among indicators can be accounted for by the general factor alone, with values > 0.7 indicating possible unidimensionality (Rodriguez et al., 2016a). Construct reliability (H) indicates the proportion of variance in a factor that is explained by its set of indicators and the likelihood that the estimated factors will replicate across studies (Rodriguez et al., 2016a). H can be calculated for general and specific factors in a bi-factor model and for the lower-order factors in a higher-order model, with values ranging from 0-1 and higher values reflecting a factor that is more replicable and well-defined by its respective indicators. Factor determinacy (FD), which measures the reliability of factor scores, is unable to be estimated accurately when using dichotomous indicators (Beauducel & Hilger, 2017; Ferrando & Lorenzo-Seva, 2018; Forbes et al., 2021) and was excluded from the current study given that a number of included indicators were dichotomous. Finaly, model selection also included evaluation of model parameters/estimates (i.e., the direction, significance, and standard errors of the factor loadings in each model; Forbes et al., 2021).

**Appendix G. Structural Validity**

Traditional model fit statistics for the four CFA models are provided in Table S2. The best-fitting factor model according to the BIC and ssaBIC was the bi-factor model. The bi-factor model also demonstrated superior fit according to CFI, TLI and RMSEA indices (CFI=0.950; TLI=0.941; RMSEA=0.040). The higher-order factor model (and the correlated-factors model by extension) demonstrated good model fit (CFI=0.926; TLI=0.920; RMSEA=0.047), whilst the one-factor model did not fit the data well (CFI=0.686; TLI=0.661; RMSEA=0.097). In contrast to traditional fit statistics, evaluation of the standardized factor loadings for each model suggested that the higher-order model was superior to the bi-factor model. For the bi-factor model, standardized factor loadings for the general factor were non-significant for all indicators. Several indicators had negative factor loadings (i.e., 6/28) and more than half (i.e., 15/28) were relatively small in magnitude (i.e., factor loadings < 0.3). In addition, there were non-significant factor loadings for 13 of the 19 indicators of internalizing (i.e., all GDS items and most K10 items). For the one-factor model, standardized factor loadings for all indicators were positive in direction but four were non-significant and eight were small in magnitude (i.e., factor loadings < 0.3). In contrast, standardized factor loadings for the general and lower-order factors from the higher-order model (and the specific factors of the correlated-factors model by extension) were all significant, positive in direction, and > 0.3 in magnitude (with the exception of a single indicator of alcohol use). The standard errors of factor loadings were also substantially lower for most indicators of the higher-order/correlated-factors models compared to the bi-factor model, indicating greater precision in estimates for the higher-order model. Standardized factor loadings, standard errors, and p-values for each of the four CFA models run using the MLR and WLSMV estimators are presented in the supplementary material (Tables S5-S12).

The imprecision of parameter estimates for the bi-factor model was further supported by model-based estimates of reliability (Table S3). The ECV value of 0.288 suggests a relatively weak general factor, accounting for only 28.8% of common variance among items in the dataset. Omega H values further revealed that general psychopathology accounted for 29.5% of the reliable variance in observed total scores after partialling out variance attributable to the specific factors. These values, in addition to the PUC value of 0.50, support the multidimensionality of the items used in the current study (i.e., ECV, Omega H, and PUC values all < 0.70). However, this multidimensionality was not reliably captured by the specific factors of the bi-factor model (i.e., ECV values ranged from 0.109 to 0.421 and omega HS was < 0.70 for the internalizing specific factor). Finally, H values indicated that the general and specific factors of the bi-factor model were well represented by their respective indicators (i.e., H values ranged from .764 to .962) but were lower than those observed for the higher-order model (i.e., H values ranged from .766 to .996).

Although traditional fit indices supported the bi-factor model, a decision was made to use the higher-order model in subsequent analyses. This decision was based on 1) evaluation of standardized factor loadings (i.e., all positive in direction and significant for the higher-order model); 2) lower standard errors of factor loadings for the higher-order model (i.e., more precise estimates of these parameters; 3) evidence of multidimensionality yet poor reliability of general and specific factors of the bi-factor model based on model-based reliability coefficients (i.e., ECV, PUC, Omega H/HS values); and 4) evidence of greater construct reliability and replicability of specific factors from the higher-order model compared to the bi-factor model (i.e., greater H values). All analyses (i.e., primary, secondary, and post-hoc) were also run with Bayesian Plausible Values (BPVs) generated for the bi-factor model. The results of these analyses are reported in the supplementary material (Appendix K and Tables S17-S21).

**Appendix H. False discovery rate correction**

Benjamini-Hochberg false discovery rate (FDR) was used to correct for multiple testing across sets of analyses, with an FDR threshold of 5% (α = 0.05). When controlling for global brain structure, one set of analyses examined associations with GMV (i.e., global cortical and subcortical GMV, regional GMV in the frontal, parietal, temporal, and occipital lobes, regional GMV in the cerebellum and hippocampus) across waves for each set of BPVs representing the four transdiagnostic symptom dimensions at baseline (i.e., 32 analyses). An additional set of analyses examined cortical thickness (i.e., average global cortical thickness and average regional cortical thickness in the frontal, parietal, temporal, and occipital lobes) across waves for each set of BPVs representing the four transdiagnostic symptom dimensions at baseline (i.e., 20 analyses). Finally, one additional set of analyses examined dementia status for each set of BPVs representing the four symptom dimensions at baseline (i.e., 4 analyses). Follow-up tests of regional associations that did not control for global brain structure were treated as a separate set of analyses for FDR correction (i.e., 24 tests of regional GMV and 16 tests of regional cortical thickness). This same approach was followed for baseline analyses controlling for global brain structure (i.e., 32 analyses for GMV and 20 analyses for cortical thickness) and not controlling for global brain structure (i.e., 24 analyses for GMV and 16 analyses for cortical thickness). Finally, analyses examining associations with BPVs generated from the bi-factor model followed the same approach to FDR correction described above.

**Appendix I. Deviations from the pre-registered analytic plan**

There were two deviations from the pre-registered analysis that should be noted. Firstly, our pre-registered analysis stated that we would examine associations with average GMV for global and regional outcome measures. However, it was decided to examine associations with total GMV in order to ensure greater consistency with previous research investigating the relationship between grey matter structure and dimensions of psychopathology (Mewton et al., 2022; Romer et al., 2023; Snyder et al., 2017). Secondly, our pre-registered analysis stated that we would re-run all models examining specific/lower-order factors whilst controlling for general psychopathology. These additional analyses were not necessary given that we found no statistically significant associations with any specific/lower-order symptom dimension.

**Appendix J. Unconditional Linear Mixed Effect Models**

We ran a series of post-hoc unconditional linear mixed effects models (i.e., without predictors included) to examine the trajectories of each brain structural outcome variable over time. All outcome measures were standardized prior to analysis and Benjamini-Hochberg false discovery rate (FDR) was used to correct for multiple testing across sets of analyses (i.e., 8 tests of GMV and 5 tests of cortical thickness). All models included random effects for the intercepts but no random effects for the slopes, which is consistent with the models used in our main analyses. Fixed effects indicated significant reductions in all brain structural outcome measures across waves (βs = -0.138 to -0.438; SEs = 0.015 to 0.047; p < 0.001), as expected in a sample of older adults. All fixed effects remained significant after false discovery rate (FDR) correction. The inter-class correlation coefficients (ICC) for these models ranged from 0.657 to 0.852, indicating substantial variability in baseline levels of total GMV and average cortical thickness between participants across all brain structural outcomes.

**Appendix K. Results from analyses using the bi-factor model**

All analyses were re-run using transdiagnostic symptom dimensions derived from a bi-factor model, following the same methodology as described in the manuscript for primary, secondary, and post-hoc analyses. General and specific factors derived from the bi-factor model were not associated with any global or regional measures of GMV across waves. Substance use was associated with greater intra-individual change in cortical thickness within the parietal lobe at wave 2 (beta = 0.007; SE = 0.003; p = 0.043); however, this association did not survive FDR correction. No other symptom dimensions were associated with change in cortical thickness over time. General psychopathology was negatively associated with total cortical GMV at baseline (beta = -3644; SE = 1588.839; p = 0.022); however, this association did not survive FDR correction. There was no evidence of an association between general psychopathology and total subcortical GMV or average cortical thickness at baseline. No specific factors were associated with any baseline measure of global brain structure (i.e., total cortical GMV, total subcortical GMV, or average cortical thickness). When controlling for global brain structure (i.e., total GMV or average cortical thickness), general and specific factors were not associated with any baseline ROI measures. When not controlling for global brain structure, general psychopathology was negatively associated with baseline GMV in the frontal (beta = -1446; SE = 633.838; p = 0.023) and temporal (beta = -9220; SE = 406.836; p = 0.024) lobes. Neither association survived FDR correction. These associations are the same as those found for internalizing in the higher-order model and likely reflect the fact that the general factor in our bi-factor model is defined primarily by internalizing items. The general factor was not associated with baseline GMV or cortical thickness in any other ROI. No specific factors were associated with any regional measure of baseline GMV or cortical thickness when not controlling for global brain structure. Finally, general and specific symptom dimensions did not predict incident dementia.

­­­­­**References**

Beauducel, A., & Hilger, N. (2017). On the bias of factor score determinacy coefficients based on different estimation methods of the exploratory factor model. *Communications in Statistics - Simulation and Computation*, *46*(8), 6144–6154. https://doi.org/10.1080/03610918.2016.1197247

Cummings, J. L., Mega, M., Gray, K., Rosenberg-Thompson, S., Carusi, D. A., & Gornbein, J. (1994). The Neuropsychiatric Inventory. *Neurology*, *44*(12), 2308–2308. https://doi.org/10.1212/WNL.44.12.2308

Desikan, R. S., Ségonne, F., Fischl, B., Quinn, B. T., Dickerson, B. C., Blacker, D., Buckner, R. L., Dale, A. M., Maguire, R. P., Hyman, B. T., Albert, M. S., & Killiany, R. J. (2006). An automated labeling system for subdividing the human cerebral cortex on MRI scans into gyral based regions of interest. *NeuroImage*, *31*(3), 968–980. https://doi.org/10.1016/j.neuroimage.2006.01.021

Dueber, D. M. (2017). *Bifactor indices calculator: a Microsoft Excel-based tool to calculate various indices relevant to bifactor CFA models*.

Ferrando, P. J., & Lorenzo-Seva, U. (2018). Assessing the Quality and Appropriateness of Factor Solutions and Factor Score Estimates in Exploratory Item Factor Analysis. *Educational and Psychological Measurement*, *78*(5), 762–780. https://doi.org/10.1177/0013164417719308

Fischl, B. (2012). FreeSurfer. *NeuroImage*, *62*(2), 774–781. https://doi.org/10.1016/j.neuroimage.2012.01.021

Folstein, M. F., Folstein, S. E., & McHugh, P. R. (1975). “Mini-mental state.” *Journal of Psychiatric Research*, *12*(3), 189–198. https://doi.org/10.1016/0022-3956(75)90026-6

Forbes, M. K., Greene, A. L., Levin-Aspenson, H. F., Watts, A. L., Hallquist, M., Lahey, B. B., Markon, K. E., Patrick, C. J., Tackett, J. L., Waldman, I. D., Wright, A. G. C., Caspi, A., Ivanova, M., Kotov, R., Samuel, D. B., Eaton, N. R., & Krueger, R. F. (2021). Three recommendations based on a comparison of the reliability and validity of the predominant models used in research on the empirical structure of psychopathology. *Journal of Abnormal Psychology*, *130*(3), 297–317. https://doi.org/10.1037/abn0000533

Goldberg, D., Bridges, K., Duncan-Jones, P., & Grayson, D. (1988). Detecting anxiety and depression in general medical settings. *BMJ*, *297*(6653), 897–899. https://doi.org/10.1136/bmj.297.6653.897

Kessler, R. ; M. D. (1994). Final version of our Non-specific Psychological Distress Scale. In *Unpublished memo*. : Survey Research Center of the Institute for Social Research, University of Michigan.

Mewton, L., Lees, B., Squeglia, L. M., Forbes, M. K., Sunderland, M., Krueger, R., Koch, F. C., Baillie, A., Slade, T., Hoy, N., & Teesson, M. (2022). The relationship between brain structure and general psychopathology in preadolescents. *Journal of Child Psychology and Psychiatry*, *63*(7), 734–744. https://doi.org/10.1111/jcpp.13513

’Muthén, L. ’ ’Muthén, B. (2018). *Mplus. The comprehensive modelling program for applied researchers: User’s guide, 5*.

Muthén, L. K. and M. B. O. (2017). Mplus User’s Guide. In *Eighth Edition.  Los Angeles, CA: Muthén & Muthén*.

Raftery, A. E. (1995). Bayesian Model Selection in Social Research. *Sociological Methodology*, *25*, 111. https://doi.org/10.2307/271063

Reise, S. P., Moore, T. M., & Haviland, M. G. (2010). Bifactor Models and Rotations: Exploring the Extent to Which Multidimensional Data Yield Univocal Scale Scores. *Journal of Personality Assessment*, *92*(6), 544–559. https://doi.org/10.1080/00223891.2010.496477

Rodriguez, A., Reise, S. P., & Haviland, M. G. (2016a). Applying Bifactor Statistical Indices in the Evaluation of Psychological Measures. *Journal of Personality Assessment*, *98*(3), 223–237. https://doi.org/10.1080/00223891.2015.1089249

Rodriguez, A., Reise, S. P., & Haviland, M. G. (2016b). Evaluating bifactor models: Calculating and interpreting statistical indices. *Psychological Methods*, *21*(2), 137–150. https://doi.org/10.1037/met0000045

Romer, A. L., Ren, B., & Pizzagalli, D. A. (2023). Brain Structure Relations With Psychopathology Trajectories in the Adolescent Brain Cognitive Development Study. *Journal of the American Academy of Child & Adolescent Psychiatry*. https://doi.org/10.1016/j.jaac.2023.02.002

Sachdev, P. S., Brodaty, H., Reppermund, S., Kochan, N. A., Trollor, J. N., Draper, B., Slavin, M. J., Crawford, J., Kang, K., Broe, G. A., Mather, K. A., & Lux, O. (2010). The Sydney Memory and Ageing Study (MAS): methodology and baseline medical and neuropsychiatric characteristics of an elderly epidemiological non-demented cohort of Australians aged 70–90 years. *International Psychogeriatrics*, *22*(8), 1248–1264. https://doi.org/10.1017/S1041610210001067

Snyder, H. R., Hankin, B. L., Sandman, C. A., Head, K., & Davis, E. P. (2017). Distinct Patterns of Reduced Prefrontal and Limbic Gray Matter Volume in Childhood General and Internalizing Psychopathology. *Clinical Psychological Science*, *5*(6), 1001–1013. https://doi.org/10.1177/2167702617714563

Yesavage, J. A., Brink, T. L., Rose, T. L., Lum, O., Huang, V., Adey, M., & Leirer, V. O. (1982). Development and validation of a geriatric depression screening scale: A preliminary report. *Journal of Psychiatric Research*, *17*(1), 37–49. https://doi.org/10.1016/0022-3956(82)90033-4
